# In silico screening and identification of deleterious missense SNPs along with their effects on CD-209 gene: An insight to CD-209 related-diseases

**DOI:** 10.1371/journal.pone.0247249

**Published:** 2021-02-26

**Authors:** Mohib Ullah Kakar, Muhammad Matloob, Rongji Dai, Yulin Deng, Kifayat Ullah, Ihsan Ullah Kakar, Ghulam Khaliq, Muhammad Umer, Zhoaib Ahmed Bhutto, Sarfarz Ali Fazlani, Muhammad Zubair Mehboob

**Affiliations:** 1 Beijing Key Laboratory for Separation and Analysis in Biomedicine and Pharmaceuticals, School of Life Sciences, Beijing Institute of Technology (BIT), Beijing, PR China; 2 Department of Biomedical Engineering, National University of Science and Technology (NUST), Islamabad, Pakistan; 3 Department of Biosciences, COMSATS University Islamabad, Islamabad, Pakistan; 4 Faculty of Veterinary and Animal Sciences, Water and Marine Sciences (LUAWMS), Lasbela University of Agriculture, Uthal, Pakistan; 5 Department of Horticulture, Faculty of Agriculture, Water and Marine Sciences (LUAWMS), Lasbela University of Agriculture, Uthal, Pakistan; 6 Department of Biochemistry and Biotechnology, University of Gujrat, Gujrat, Pakistan; University of Texas Rio Grande Valley, UNITED STATES

## Abstract

DC-SIGN receptor articulated by macrophages and dendritic cells is encoded by *CD209* gene and plays a role to activate and proliferate the T-lymphocytes in response of virus attack. The dysfunctional activity of DC-SIGN receptor because of missense SNPs can lead to cause dengue haemorrhage fever, HIV-1 infection etc. Out of 11 transcripts of *CD209*, all missense SNPs of canonical transcript were retrieved from Ensembl database and evaluated by their deleteriousness by using Polyphen-2, PMut, SIFT, MutPred, PROVEAN and PhD-SNP together with stimulation of its complete 3D structure. 10 nsSNPs were chosen depending on both the significance value of nsSNP and their prediction among SNPs evaluating servers which are based on different algorithms. Moreover, the position and native role of 10 nsSNPs in wild 3D model has been described which assist to acknowledge their importance. This study urges the researcher’s community to experimentally validate these SNPs and their association in causing the diseases like dengue fever, Tuberculosis etc.

## Introduction

*CD209* gene encodes dendritic cell-specific intracellular adhesion molecule-3 grabbing non-integrin (DC-SIGN) receptor which is articulated by macrophages and dendritic cells [1–3] that participant in innate immune response. DC-SIGN is a soluble transmembrane protein which belongs to C-type lectin protein family and possesses three renown domains; N-terminal cytoplasmic domain, neck region (encompassing octa 23 amino acids repeats) and a C-type lectin domain (C-terminal) [4]. CD209 interacts with the surface mannose or oligosaccharides moieties of extraneous intruders, including HIV-1, Ebola virus, Cytomegalovirus, and Dengue virus, resulting in T-lymphocyte activation and proliferation which in turn activate the immune response cascade [5, 6]. Several studies have described an association of single nucleotide polymorphism (SNPs) and human diseases. As SNPs are the prevalent form of mutation in the human genome and have been reported in coding, non-coding as well as in intergenic zones [7, 8]. Coding SNPs are either synonymous, having a nucleotide transition that does not bring about the amino acid shift, or non-synonymous (nsSNPs), a nucleotide transition concordant with the amino acid shift. nsSNPs. The latter ones are more effective and can potentially effect protein stability, charge, solubility, structure and function. A small fraction of nsSNPs is deleterious which are always been a great interest for scientific community as being associated to cause various complex diseases in humans [9–11].

Many nsSNPs of non-coding regions of CD209 have been investigated previously, which were implicated to cause different diseases [12–17]; for instance, promoter region SNP -939 G/A was found to trigger tuberculosis in Indonesian and African populations [18, 19]. In addition, one more mutation -336 G/A in promoter region was reported to contribute [20–22] in parental HIV-1 infections in the European-American population, dengue hemorrhagic fever in Thailand and Taiwan population [23] and Kawasaki disease in Chinese population [24]. Despite of promoter region, a few mutations are also reported in 3’UTR regions such as rs2287886 and rs7248637, associated with colorectal cancer [25] and severe form of tick-borne encephalitis in the Russian population [5]

Based on these infectious threats posed by the nsSNPs reported in non-coding regions, the present study is aimed to locate nsSNPs in coding regions of CD209 and to narrow down the list of deleterious nsSNPs by using computational tools. This advantageous study will help to screen future genotypes and identify the notorious variants in CD209 which can exacerbate aforementioned diseases.

## Methodology

### Dataset used for missense SNPs annotation

A list of missense SNPs of CD209 was retrieved from Ensembl database which includes the reported SNPs of dbSNP and Cosmic database Out of 11 transcripts with different length, transcript having longest length known as the canonical transcript was selected and further dig to retrieve all missense SNPs.

### Prediction of damaging SNPs

The functional effect of all missense SNPs was predicted by the enlisted software. Table 1 summarizes all servers used in this study to estimate deleterious impact of missense SNPs and to design CD-209 structure.

**Table 1:** Summary of all software used to find out harmful missense SNPs and their impact on CD-209 model.

Software	Category	Input method	Algorithm	Score
**PhD-SNP**	function prediction	Protein sequence and substituted amino acid along with position	SVM-based method using protein sequence and profile information	No define category
**SIFT**	function prediction	Protein sequence, db SNP id, protein Id	uses sequence homology, predicts whether an amino acid substitution affects protein function based on sequence homology and the physical properties of amino acids	Score ranges from 0 to 1, where < = 0.05 is damaging and >0.05 is tolerated
**PolyPhen-2**	function prediction	Protein sequence, db SNP id, protein Id	Uses sequence conservation and structure to model location of amino acid substitution, Swiss-Prot and TrEMBL annotation	Score ranges from 0 to 1, where < = 0.05 is benign, and >0.05 is damaging
**MutPred**	function prediction	Protein id, PS, or multiple sequence alignment	Protein sequence-based model using SIFT and a gain/loss of 14 different structural and functional properties	Score ranges from 0 to 1, where 0 is polymorphism and high scores are predicted to be deleterious/disease-associated
**PROVEAN**	function prediction	protein sequence	Uses an alignment-based score approach to generate predictions not only for single amino acid substitutions, but also for multiple amino acid substitutions, and in-frame insertions and deletions	the default score threshold is currently set at -2.5, in which >-2.5 is neutral, and <-2.5 is deleterious
**PMUT**	function prediction	PS and AAS, dbSNP, Uniprot or PDB ID of protein	Based on the application of neural networks which uses internal databases, secondary structure prediction, and sequence conservation	Score ranges from 0 to 1, where <0.50 is neutral and >0.50 is disease associated
**I-TASSER**	Structure prediction	protein sequence	identifies structural templates from the PDB by multiple threading approach LOMETS, with full-length atomic models constructed by iterative template-based fragment assembly simulations	C-score ranges from -5 to 2, greater the score means higher the global topology
**ModRefiner**	3D model refinement	pdb model of protein	uses an algorithm for atomic-level, where conformational search is guided by a composite of physics- and knowledge-based force field	
**I-Mutant **	protein stability	protein pdb model, protein sequence	SVM based predictor for protein stability changes upon single point protein mutation starting from structural informations	
**ConSurf**	estimating the evolutionary conservation of amino	protein sequence	Carries out a search for close homologous sequences. A multiple sequence alignment (MSA) of the homologous sequences is constructed, Position-specific conservation scores are computed using the empirical Bayesian	

First six servers are SNPs evaluating software used to check the deleteriousness of missense SNPs. These softwares used different algorithm at backend and predict one SNPs as damaging or benign by giving one score to each SNPs. CD-209 model generated by I-TASSER is further refined by ModRefiner server, and amino acid conservation at a specific place is determined by conservation score predicted by Consurf server.

**Polyphen-2** tool is used to predict the potential effect of the amino acid substitution i.e., damaging or benign by utilizing structural and evolution characteristics. The Polyphen-2 score ranges from 0 to 1. If the score is near to 1, missense SNP comes under probably damaging [26].

**PMut** predicts the severity (pathological or neutral) of the substituted amino acid in a particular position. PMut relies on sequence alignment and structural factors by using the feed-forward neural network. The output file is comprised of the confidence index and binary prediction of “neutral” versus “pathological” [27].

**SIFT** (Sorting Intolerant From Tolerant) web tool uses the protein database by PSI-BLAST and collects functionally related protein sequences. Subsequently, by sequence alignment, it finds out the probability of an amino acid at a particular position. The scores <0.05 are considered as in-tolerated whereas scores >0.05 are taken as tolerated [28].

**MutPred** is used to predict the changes in structural features and functional site due to amino acid substitution. MutPred builds upon the established SIFT method and a gain or loss of 14 different functional and structural properties. In MutPred results, the G-value ranges from 0 to 1. Higher the G-value, greater will be the effect of amino acid substitution on structure and function of protein(s) [29].

**PROVEAN** uses the primary sequence of target protein and its homologs are searched via sequence alignment by BLAST in NCBI nr-database. The result of PROVEAN is measured as PROVEAN score whereas cut-off value is -2.5. amino acid substitution with PROVEAN scores greater than -2.5 is considered deleterious [30].

**PhD-SNP** (Predictor of human Deleterious Single Nucleotide Polymorphisms) is an SVM-based classifier. The output result is tabulated and mentioning the nature of change either deleterious or neutral [31].

### 3D structure prediction of CD209 protein

The 3D structure of wild type and mutated proteins was simulated by using I-TASSER based on iterative-threading approach [32]. Since the crystal structure of C-lectin domain of CD209, involved in recognition and binding to sugar moiety present on the surface of pathogens is available in PDB database, however, yet its complete structure in not resolved. So, a complete 3D model is designed by I-TASSER server.

### Energy minimization and validation of wild-type and mutant models

Wild-type and all mutated models were refined by ModRefiner which refine the structure to atomic levels and remove worse psi and phi angles [33]. These minimized models were evaluated by RAMPAGE used to form the Ramachandran plot, important to check protein quality.

### Predicting the stability change of mutated models

**I-MUTANT 3.0** is used to predict protein stability during point mutation. This tool retrieves data from ProTherm, a database providing experimental proved free energy change of protein stability upon point mutation. The input file is comprised of protein sequence along with new residue and position number for obtaining the free energy change [34].

### Conservation analysis

Evolutionary conservation of residues features the historical importance in a specific place and any alternation can disturb the normal function of proteins. To calculated the evolutionary conservation of amino acids, the ConSurf server was used which estimate the preservation sequence homology [35]. It shows the conservation score from 1 to 9, where residue with maximum score i.e., 9 is highly conserved. It only requires the FASTA sequence of the gene.

## Results and discussion

### Missense SNPs retrieval and annotation

Canonical transcript of CD209 encompassed total 693 SNPs, including 27 stop gained, 17 frameshift, 137 synonymous SNPs and 227 missense SNPs. We selected the missense SNPs which were further evaluated by SNPs evaluating online servers. These servers are used to identify and differentiate the deleterious missense SNPs from benign. The Polyphen-2 categorized 135 missense SNPs out of 227 as possibly or probably damaging which counted 60% of total number of SNPs while remaining 40% were represented as benign. According to neural network based PMut, 167 SNPs were neutral, i.e., they will not damage the protein structure and function, and only 60 SNPs met the criteria of being deleterious. Similarly, according to SIFT prediction, 127 damaging missense SNPs weighed 56% of total number of SNPs and 100 candidates were identified as normal. PROVEAN server that uses the alignment-based prediction of substitution represented 68 SNPs (28%) under damaging category whereas 78% (159 SNPs) were shown as neutral. Likewise, 82 and 28 missense SNPs were concluded as deleterious by using algorithm of the PhD-SNP and MutPred respectively. As all these online server uses different models at backend to predict the pathogenicity of SNPs, so varying number of damaging SNPs were predicted by each server S1 Table. At end, there were a total 27 SNPs which were predicted pathogenic by all the servers Table 2.

**Table 2:** List of 27 most deleterious missense SNPs along with their software scores.

Mutations	PhD-SNP	PhD-SNP score	PolyPhen-2	PolyPhen-2 score	PMut	PMut score	PROVEAN	PROVEAN score	SIFT	SIFT score	MutPred	MutPred score
D320Y	Dis	7	Dam	1	Patho	0.6189	Dele	-8.03	Dam	0.006	Patho	0.838
D331H	Dis	5	Dam	1	Patho	0.7431	Dele	-6.36	Dam	0	Patho	0.828
D366A	Dis	7	Dam	1	Patho	0.7026	Dele	-7.18	Dam	0.001	Patho	0.853
D366N	Dis	5	Dam	1	Patho	0.6864	Dele	-4.49	Dam	0.001	Patho	0.78
E299K	Dis	7	Pro. Dam	0.999	Patho	0.62	Dele	-3.78	Dam	0	Patho	0.885
E347K	Dis	7	Dam	0.998	Patho	0.6935	Dele	-3.39	Dam	0.011	Patho	0.787
E358A	Dis	2	Dam	1	Patho	0.5675	Dele	-5.27	Dam	0.009	Patho	0.759
E358K	Dis	2	Pro. Dam	1	Patho	0.67	Dele	-3.51	Dam	0.01	Patho	0.681
F302V	Dis	3	Dam	0.995	Patho	0.6833	Dele	-6.28	Dam	0.001	Patho	0.723
G265R	Dis	6	Poss. Dam	0.953	Patho	0.63	Dele	-6.24	Dam	0.01	Patho	0.614
G317E	Dis	8	Dam	1	Patho	0.7982	Dele	-7.23	Dam	0	Patho	0.861
G332S	Dis	7	Dam	1	Patho	0.6919	Dele	-4.91	Dam	0.008	Patho	0.767
G346E	Dis	5	Dam	1	Patho	0.7274	Dele	-6.92	Dam	0.001	Patho	0.679
G346R	Dis	6	Dam	1	Patho	0.7092	Dele	-6.92	Dam	0.004	Patho	0.678
L291F	Dis	7	Dam	1	Patho	0.8101	Dele	-3.64	Dam	0	Patho	0.63
L318F	Dis	7	Dam	1	Patho	0.6951	Dele	-3.64	Dam	0	Patho	0.586
L318P	Dis	8	Dam	1	Patho	0.7982	Dele	-6.37	Dam	0	Patho	0.899
M316T	Dis	5	Dam	1	Patho	0.5071	Dele	-5.04	Dam	0.001	Patho	0.532
P348L	Dis	6	Pro. Dam	1	Patho	0.7	Dele	-8.98	Dam	0	Patho	0.877
R251C	Dis	7	Dam	0.999	Patho	0.51	Dele	-5.61	Dam	0.015	Patho	0.509
S280F	Dis	9	Pro. Dam	1	Patho	0.8	Dele	-4.98	Dam	0	Patho	0.841
S296I	Dis	5	Dam	0.971	Patho	0.7546	Dele	-5.1	Dam	0	Patho	0.734
S308F	Dis	5	Poss. Dam	0.886	Patho	0.52	Dele	-4.62	Dam	0.03	Patho	0.636
S333L	Dis	5	Pro. Dam	1	Patho	0.75	Dele	-5.31	Dam	0	Patho	0.808
W260C	Dis	6	Dam	1	Patho	0.829	Dele	-11.77	Dam	0	Patho	0.922
W315R	Dis	8	Dam	1	Patho	0.7982	Dele	-12.71	Dam	0	Patho	0.859
W343G	Dis	8	Dam	1	Patho	0.829	Dele	-11.61	Dam	0.002	Patho	0.896

These 27 missense SNPs were called most deleterious by all the servers, which assign a special score to each SNPs depending on the algorithm used to predict functional impact. We used short form of words, such as Dis = Disease, Dam = Damaging, Pro. Dam = Probably damaging, Poss. Dam = Possibly damaging, Patho = Pathogenic, Dele = Deleterious. As all these servers have different specificity and sensitivity to detect damaging SNPs, we have assigned 25% weightage sore to PMut, MutPred and PROVEAN result, where 12.5% weightage is given to PolyPhen-2 and SIFT. In criteria we did not include the PhD-SNP server because it does not have any define cut-off value to differentiate benign from damaging missense SNPs.

Because SNPs servers used different scale to generate scores value of SNPs along with prediction, to better utilize the predicted scores, we adopted a way to build a composite quantitative score that objectively combines the scores value into single value that can further be used to rank the various nsSNPs. Two methods were employed for getting composite score, which included; 1) performing a principal component (PC) analysis (PCA) method developed by Wijndaele and colleagues [36]; 2) zero-phase components analysis” (ZCA), developed by Bell and Sejnowski [37]. PCA analysis of Wijndaele and colleagues includes two-step process A) identifying the PCs with eigenvalues greater than 1; and B) summing the varimax rotated PC scores, and the analysis of PCA followed by varimax rotation is known as PC factor analysis (PCFA). We slightly modified the PCs selection stage not only to explain the PCs explaining > 80% the total variance but also on eigenvalues greater than 1. The first, second and third PCs were showing percent variance of 52.1, 20.7 and 11.4, respectively, so under PCFA1, the first two PCs were weighted by their percent variance, while under PCFA2, the first three PCs were selected. ZCA was also used to obtain a composite quantitative score, aiming to whitening the data i.e., decorrelating, and more recently, the ZCA approach has been used quite heavily in bioinformatics and omics analyses, especially in the work of Strimmer and colleagues [38–40]. Out of 5 best known whitening approaches, Kessy et al. (2018) [41] suggested that the ZCA-cor whitening matrix (where “-cor” refers to a ZCA derived from a correlation matrix) had the best properties of decorrelating the data while being maximally similar to the original variables.

From these new composite scores, p-values based on a two-sided hypothesis test using the standard normal distribution (i.e., a two-tailed z-test) was obtained followed by rubric in Benjamini et al. (2001) [42] for controlling the Benjamini-Hochberg (BH) false discovery rate (FDR) at 0.05, which is a current way to account for multiple hypothesis testing but is not nearly as conservative as the Bonferroni procedure of dividing the p-values by the number of tests [43].

Results for the top 20 ranked p-values, where the lowest p-value receives the highest rank of 1, were reported in Table 3. Following the rubric of Benjamini et al. (2001) of starting from the bottom of the list and proceeding upward while comparing the FDR-interval value to the corresponding p-value, we declared significance starting at the first instance where the FDR-interval value is greater than the corresponding p-value. For the PCFA1 and PCFA2 scores, the top 4 SNPs gave rise to significant results on controlling for multiple testing by the BH FDR. Conversely, no SNP for the ZCA-cor scores remained significant. Further, there was very little overlap between the top 20 SNPs for PCFA1 and PCFA2 on the one hand in comparison to those for ZCA-cor on the other.

**Table 3:** Results for PCFA1, PCFA2, and ZCA-cor composite scores.

Mutation	PCFA1	p-value*	Mutation	PCFA2	p-value	Mutation	ZCA-cor	p-value	rank	FDR_int
W315R	4.63	**1.86E-06**	W315R	4.47	**3.99E-06**	W258R	-2.5	0.0063	1	0.00011
W343G	4.19	**1.38E-05**	W343G	4.06	**2.42E-05**	E299K	2.48	0.0065	2	0.00022
W260C	3.98	**3.52E-05**	W260C	3.86	**5.63E-05**	E347K	2.41	0.008	3	0.00033
C256Y	3.56	**0.0002**	C256Y	3.48	**0.0003**	S280F	2.29	0.011	4	0.00044
P348L	2.87	0.002	P348L	2.84	0.0023	R198Q	2.19	0.0142	5	0.00055
D320Y	2.64	0.0042	D320Y	2.62	0.0044	D279V	-2.19	0.0143	6	0.00066
G317E	2.46	0.0069	G317E	2.46	0.007	R221Q	2.18	0.0145	7	0.00077
W258R	2.38	0.0088	D366A	2.31	0.0104	H40R	-2.16	0.0155	8	0.00088
D366A	2.3	0.0106	L318P	2.14	0.016	M166L	2.09	0.0185	9	0.00099
L318P	2.12	0.0168	W258R	2.13	0.0165	K285Q	2.07	0.019	10	0.0011
G346R	2.06	0.0196	G346R	2.09	0.0185	G55E	-2.06	0.0196	11	0.00121
G346E	1.92	0.0273	S307T	-2.04	0.0207	N276D	-2.06	0.0198	12	0.00132
S307T	-1.92	0.0277	G346E	1.96	0.0252	I281V	2.04	0.0208	13	0.00143
G265R	1.79	0.0365	G265R	1.82	0.0347	I67L	1.99	0.0233	14	0.00154
S280F	1.72	0.0431	S280F	1.76	0.0388	P42L	-1.97	0.0243	15	0.00165
D331H	1.7	0.0445	D331H	1.75	0.04	E191K	1.95	0.0255	16	0.00176
R275W	1.7	0.0445	R275W	1.75	0.04	V293I	1.94	0.0264	17	0.00187
R251C	1.68	0.0461	R251C	1.74	0.0413	W152R	1.91	0.0279	18	0.00198
I146T	-1.5	0.0672	I146T	-1.59	0.0555	R73K	1.88	0.03	19	0.00209
G332S	1.41	0.0795	D367N	-1.54	0.0613	L291F	1.84	0.0326	20	0.0022

*p-values remaining significant while controlling for the FDR at 0.05 are bolded.

**Fig 1:**
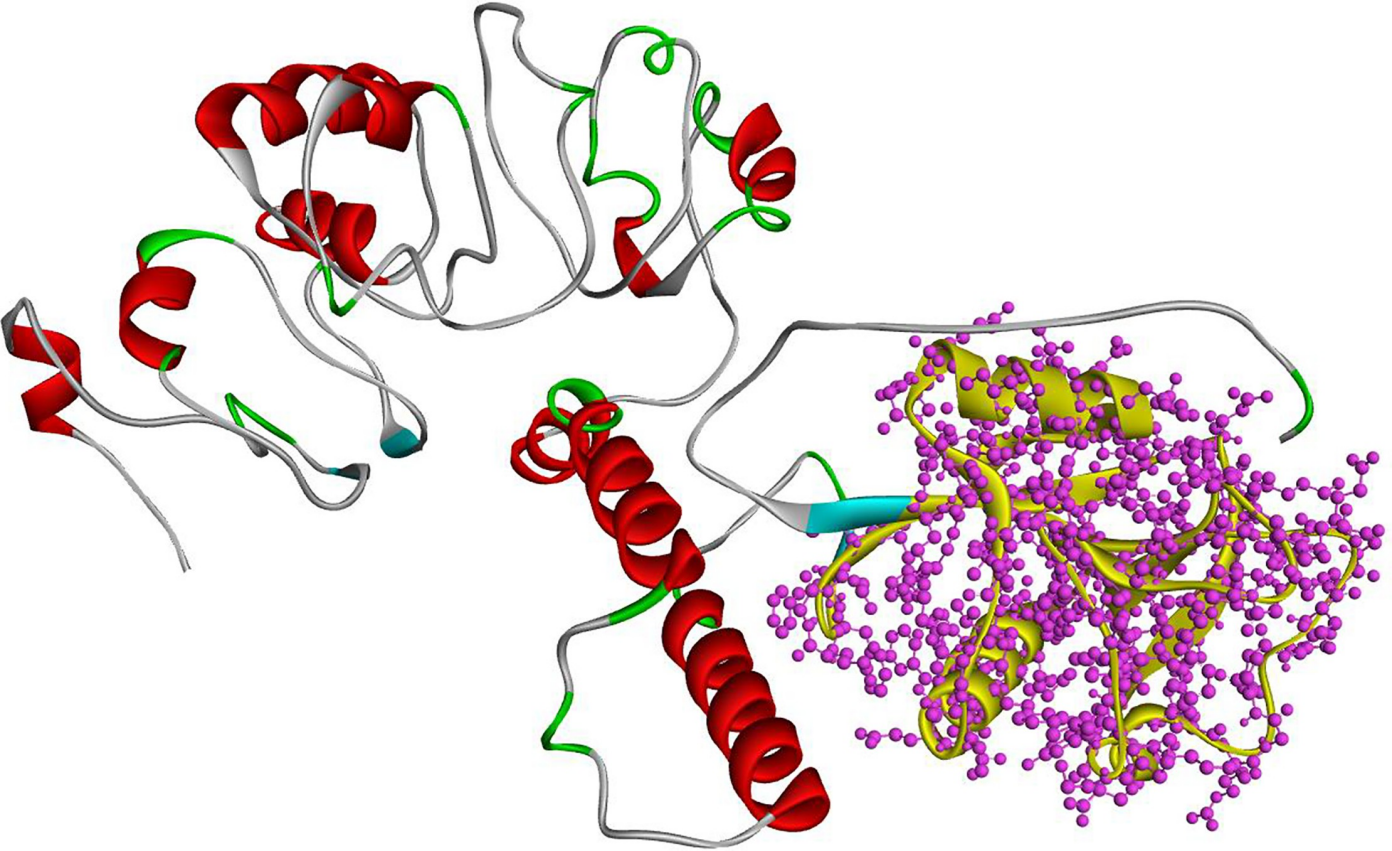
Receiver Operator Characteristic (ROC) curves for 3 logistical regression models. Full model (blue curve): PCFA2 and ZCA-cor composite scores are predictors. PCFA2 Model (red curve). ZCA-cor Model (green curve).

Lastly, a dichotomous variable called “Consensus”, scored 1 if the SNP was one of the 27 (predicted deleterious), 0 otherwise, was created which followed by logistic regression analysis as the outcome and the PCFA1, PCFA2, and ZCA-cor scores as the predictors. Using the “drop 1” sequential variable selection method [44], a best model with just PCFA2 and ZCA-cor as predictors was finalized. Using this model, a receiver operator characteristic (ROC) curve analysis was performed to compare the relative performance of PCFA2 and ZCA-cor at predicting the Consensus variable (Fig 1; Table 4). The ROC curve analysis merely shows that these composite quantitative scores are good predictors of the dichotomous Consensus variable, so we can use these models to predict the deleterious missense SNPs. The real test, however, regarding their efficacy and utility would be in regard to predicting a dichotomous disease susceptibility or resistance variable. For instance, C256Y mutation is ranked 4th in PCFA1 and PCFA2 but MutPred server predicted it neutral and similarly W258R is ranked 8th in PCFA1 but it was called damaging only by PROVEAN. This statistical analysis helps us to rank the nsSNPs according to their significance scores, but we judged this ranking according to prediction of servers also. Interestingly, out of top 20 ranked nsSNPs by PCFA2, 14 mutations were exactly those present in Table 2. i.e., unanimously selected. We decided to proceed for further biological analysis by selecting those top 10 mutations that secured high rank in PCFA2 (best model in ROC) and also predicted pathogenic by all servers. These mutations were W315R, W343G, W260C, P348L, D320Y, G317E, D366A, L318P, G346R and G346E.

**Table 4:** Area under the curve per model with their 95% confidence intervals.

Model	AUC	95% Lower Bound	95% Upper Bound
Full Model	0.9974	0.9974	1
PCFA2 Model	0.9735	0.9735	0.9922
ZCA-cor Model	0.76	0.76	0.8579

**Fig 2:**
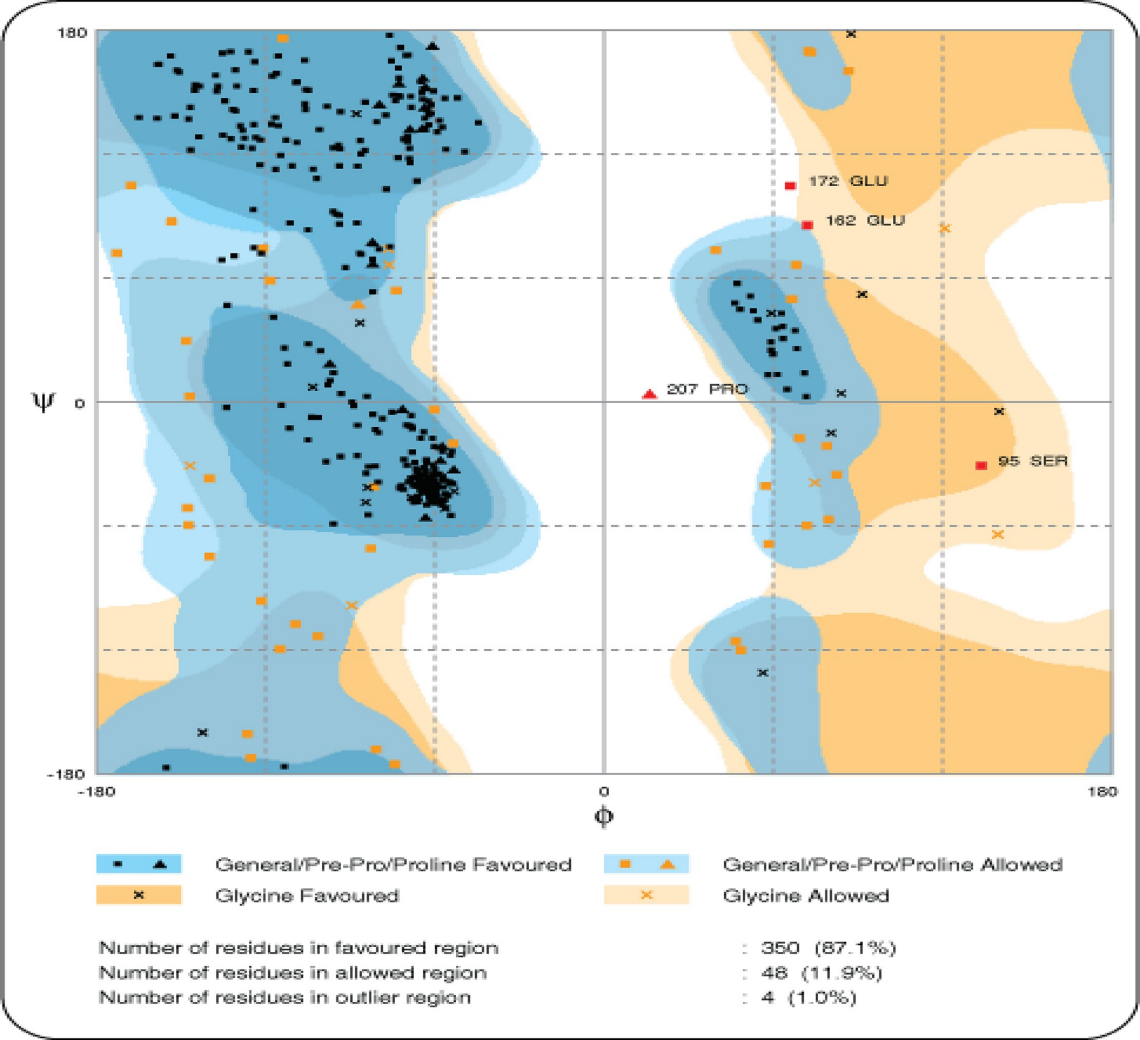
Complete 3D structure simulated by I-TASSER. Complete CD209 structure is colored according to secondary protein structures. In this structure, red color shows the location of alpha-helixes and turquoise of beta-sheets. The one part with yellows color represents the C-Lectin domain involved in ligand binding.

**Fig 3:**
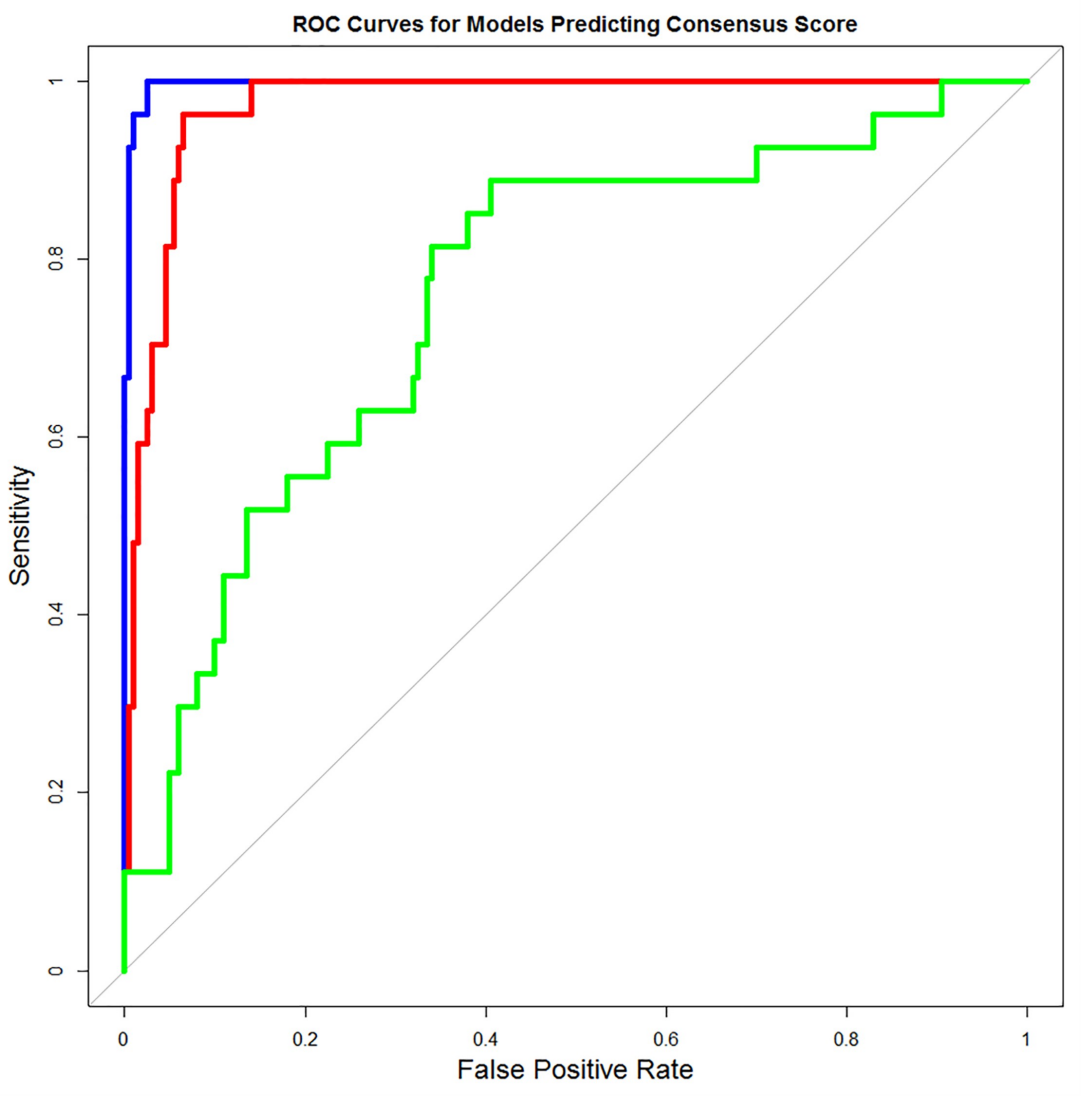
Ramachandran plot of complete model. This model shows the number of residues in the favored, allowed and outlier region. As mostly glycine has two hydrogen atoms attached to its side chain so if it lies in outlier region, it does not affect the overall 3D structure.

I-TASSER generated five structures having a C-score (confidence score). C-score is based on the significance of threading template alignments and the convergence parameters of the structure assembly simulations. C-score typically ranges from -5 to 2 and 3D models with low C-score were considered as the best model. Out of five predicted models, a model having C-score -2.51 was selected, and its quality was assessed by ERRAT server. Moreover, I-TASSER output also included the ligand binding site, which constituted residues 311, 347, 349, 350, 358, 365, 366, 367 and 373, by using the GQ2 (6-O-alpha-D-glucopyranosyl-4-O-sulfo-alpha-D-glucopyranose) as a ligand. Interestingly, Cys256 and Cys284 formed a disulfide bridge with Cys267 and Cys377, respectively, in 3D structure and considered important to maintain the 3D globular structure. After the refinement, a total of 350 residues (87.1%) resided in the favored region whereas 48 (11.9%) and only 4 (1%) were in allowed and outlier regions, respectively. 3D structure of CD209 protein is shown in Fig 2 along with the results of Ramachandran plot Fig 3.

### Effects of SNPs on protein stability

Protein stability is a net balance of forces which determine whether a protein will be in native folded form or denatured. A ΔΔG prediction by I-Mutant showed that 8 nsSNPs decreased protein stability (ΔΔG < 0), whereas remaining 2 variants can increase protein stability (ΔΔG > 0). In addition, the solubility, charge, and polarity analysis were also carried to check the chemical properties of substituted residues. Out of all, 9 substituted residues had changed the solubility factor by having hydrophilic, hydrophobic and neutral character, showing reverse characteristics of native residue. In case of charge analysis, 6 residues highlighted where replacement to other residue can alter charged-on protein by having positively, negatively or uncharged feature, and 5 residues were mutated to entities having polar or non-polar behavior i.e., inverse of native residue Table 5.

**Table 5:** Effect of deleterious SNPs on protein stability along with their solubility, charge and polarity properties.

Rank	Mutations	I-mutant	Solubility		Charge		Polarity	
1	W315R	Decrease	Hydrophobic	Hydrophilic	uncharged	positively charged	Non-Polar	Polar
2	W343G	Decrease	Hydrophobic	Neutral	uncharged	uncharged	Non-Polar	Non-Polar
3	W260C	Decrease	Hydrophobic	Hydrophobic	uncharged	uncharged	Non-Polar	Non-Polar
4	P348L	Decrease	Neutral	Hydrophobic	uncharged	uncharged	Non-Polar	Non-Polar
5	D320Y	Increase	Hydrophilic	Neutral	negatively charged	uncharged	Polar	Polar
6	G317E	Increase	Neutral	Hydrophilic	uncharged	negatively charge	Non-Polar	Polar
7	D366A	Decrease	Hydrophilic	Hydrophobic	negatively charged	uncharged	Polar	Non-Polar
8	L318P	Decrease	Hydrophobic	Neutral	uncharged	uncharged	Non-Polar	Non-Polar
9	G346E	Decrease	Neutral	Hydrophilic	uncharged	negatively charged	Non-Polar	Polar
10	G265R	Decrease	Neutral	Hydrophilic	uncharged	positively charged	Non-Polar	Polar

### Phylogenetic conservation

Conservation analysis is performed to monitor the conservation of residue at the position than non-conservative site. Amino acids found conserved in proteins are considered essential for protein activity and their mutation can abolish the protein activity completely. Top 10 ranked missense SNPs were highly conserved with a score between 7 and 9 Table 6. Evolutionary conserved residues play an important role either in formation of ligand domain, maintenance of core region or involved in 3D structure formation. Together with it, we also screened the effect of missense SNPs on protein structure by Ramachandran plot analysis. Normally, good quality proteins adjust their psi and phi angles in order to get a compact 3D form and their most residues lie in favourable or allowed regions, having small number of outliers. For all 10 missense SNP, we designed the mutated models and run through the RAMPAGE software which had shown that different number of residues lie in favourable, allowed and outlier regions Table 6.

**Table 6:** Ramachandran analysis of all the mutated models in addition to evolutionary conservation score predicted by ConSurf.

Missense SNPs	Ramachandran Plot Analysis			ConSurf Conservation Score
	Number of residues in favoured region	Number of residues in allowed region	Number of residues in outlier region	
W315R	336 (83.37%)	38 (9.43%)	29 (7.20%)	7
W343G	336 (83.37%)	38 (9.43%)	29 (7.20%)	7
W260C	336 (83.37%)	38 (9.43%)	29 (7.20%)	7
P348L	335 (83.33%)	39 (9.70%)	28 (6.97%)	8
D320Y	336 (83.58%)	37 (9.20%)	29 (7.21%)	9
G317E	335 (83.33%)	38 (9.45%)	29 (7.21%)	8
D366A	336 (83.37%)	38 (9.43%)	29 (7.20%)	9
L318P	333 (83.08%)	37 (9.20%)	31 (7.71%)	8
G346E	336 (83.37%)	38 (9.43%)	29 (7.20%)	8
G265R	336 (83.58%)	37 (9.20%)	29 (7.21%)	8

Plot software to assess the impact of each SNP on overall protein structure. Many deleterious SNPs changes the number of amino acids in outlier region, which mean that when substituted they change conformation in CD-209 model, results in psi- and phi angle disruption. Moreover, high Consurf conservation score means that the respective residue is highly conserved at that position, and interestingly, majority of these damaging SNPs are conserved in CD-209.

The C-lectin domain of CD209 is the core site for recognition and binding of carbohydrate moieties of pathogens and our results suggested that mostly deleterious nsSNPs were annotated in C-lectin domain only, where wild type residues can develop interactions with ligands as well as may involve maintaining the conformation. we also assessed the interactions developed by substituted residues with neighbouring amino acids.

#### D320Y, D366A

**Fig 4:**
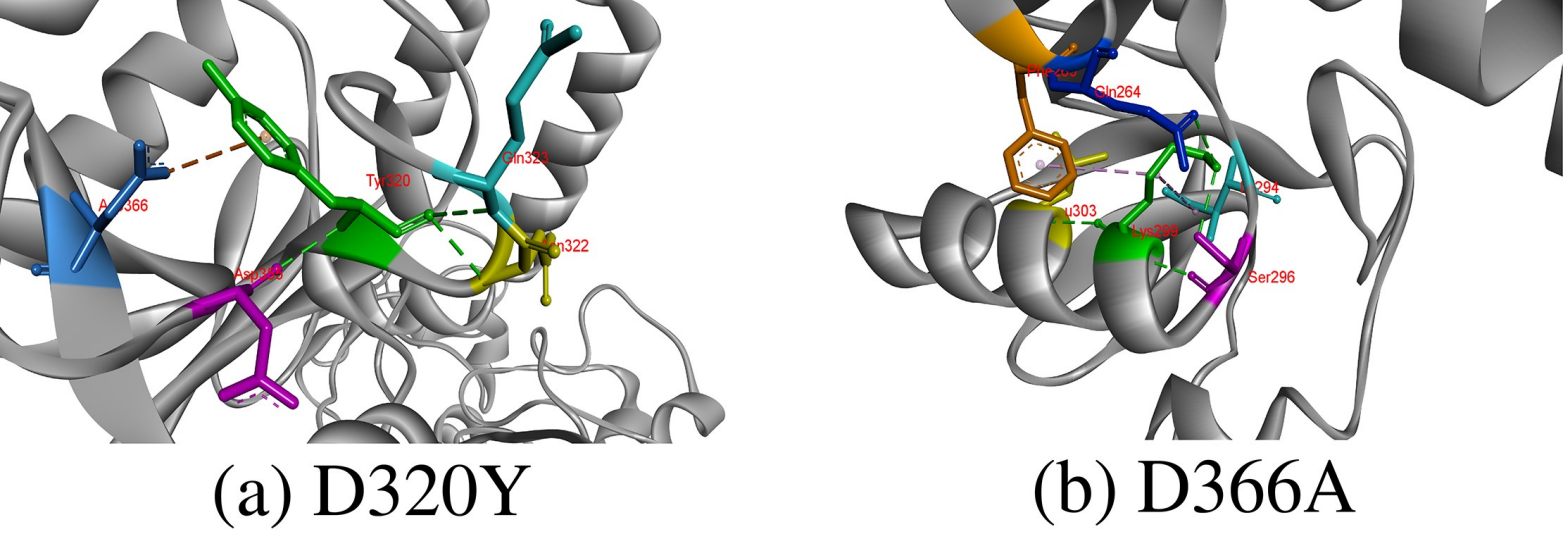
Hydrogen bonds and other interaction created by substituted amino acid at 320 and 366 positions in CD-209. The substituted amino acids are represented in green color, which form interaction with other surrounding residues colored differently. Moreover, green color is also selected to indicate hydrogen bond, whereas other bond colors represents other hydrophobic or electrostatic interactions.

Wild-type aspartic acid is a negatively charged and polar amino acid, so it prefers to be present on protein surface but can also be present in buried area of protein where it involves forming salt-bridges by interacting with positively charged amino acids and creates stabilized hydrogen bonds that can be important for protein stability. Importantly, aspartic acid residues at 320 and 366 position were highly conserved with Consurf score of 9, which indicates that substitution at these positions will results in harmful effect on proteins structure and function. Asp320 was contributing to CD-209 structure stability by forming hydrogen bonds with Asp355, Asn322, Gln323 and Gly325, and its replacement to hydrophobic tyrosine at 320 position results in breakage of hydrogen bond with Gly325 and formation of an electrostatic interaction with Asp366. Missense SNPs that result in change of Asp366 with alanine was also predicted deleterious by our study. Asp366 forms a hydrogen bond with Pro348, and when we replaced Asp366 with alanine, it caused breakage in hydrogen bonds with Pro348 Fig 4.

#### G265R, G317E, G346E

**Fig 5:**
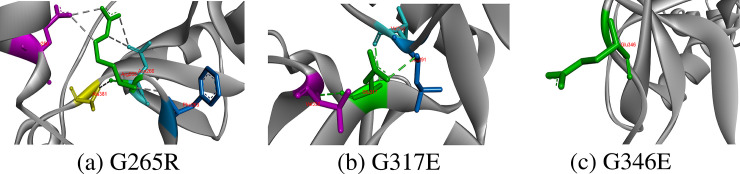
Replacement of Glycine at 265, 317 and 346 positions with respective residues and their hydrophobic and hydrogen bonds interactions.

Hydrophobicity and small size of Glycine make it unique residue in protein because torsion angles formed by glycine are unusual and can only be formed by glycine. It contains only hydrogen atom on its side chain, thus providing conformational flexibility to CD-209 protein. It mostly resides in loops and tight turns of proteins where other amino acids are forbidden; therefore, wild-type glycine residues showed conservation in CD-209 structure with Consurf score 8 (highly conserved). Gly265 formed two hydrogen bonds with CD-209 residues Phe263 and Ala381. Glycine changing with amino acid larger in size disrupts conformation of protein. Both hydrogen bonds were also established by replaced Arg265, which also developed two extra hydrogen bonds with each Glu260 and Asn266. Two hydrogen bonds constituted by Gly317 with Val292 and Val330 were not only retained by substituted glutamic acid but also it constituted one extra hydrogen bond with Leu291; thereby, glycine replacement to positive charged hydrophilic glutamic acid would disturb the torsion angles. In addition, no hydrogen bond was observed formed by Gly346 Fig 5.

#### L318P, P348L

**Fig 6:**
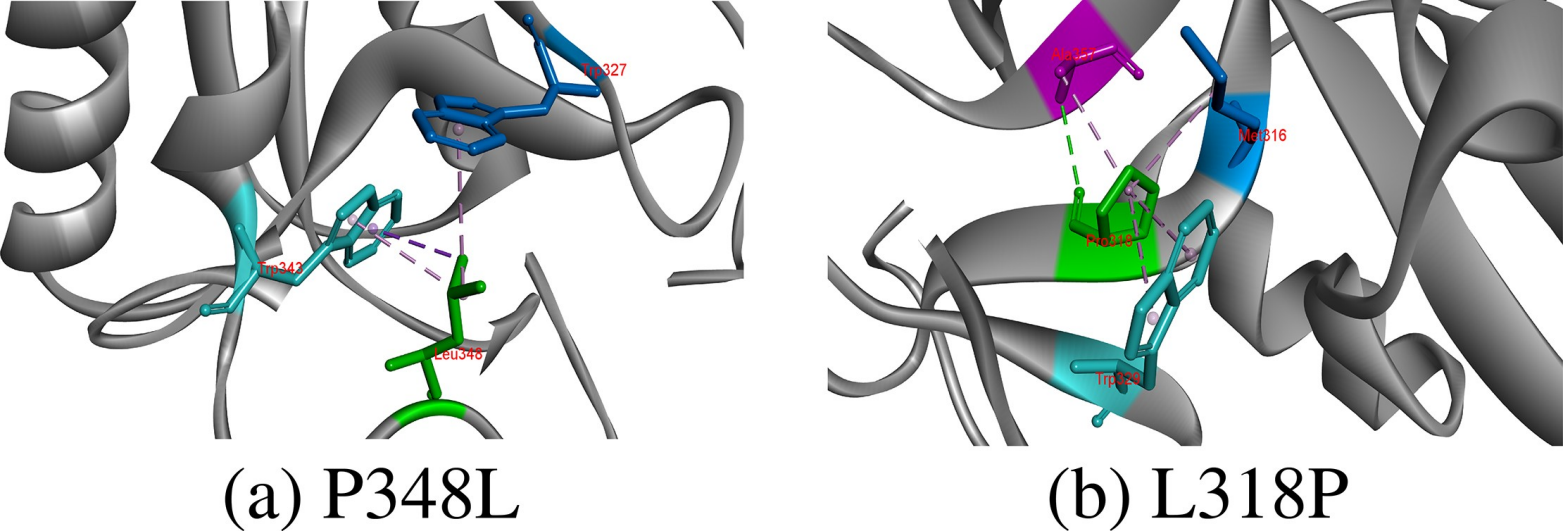
Hydrogen bonds, hydrophobic and electrostatic interaction of substituted amino acids at 318 and 348 positions.

Leucine is hydrophobic residue and found in buried cores of proteins, where it rarely directly involves in protein function because of non-reactive side chain and helps in recognizing substrates molecules. Leucine residue at 318 showed Consurf score of 8, proposing its conservation at these positions. Leu318 was involved in making two hydrogens bonds with Met316 and Ala357 and seven hydrophobic interactions with Trp329, Met316, Leu335, Ala357, Trp327 and Trp364. Although hydrophobic in nature, proline318 substitution resulted in breakage of one hydrogen bond with Met316 and other hydrophobic interactions with Leu335, Trp327 and Trp364 Fig 6.

The proline is the only secondary amine, whose side chain is connected to protein backbone twice. In protein structures, proline introduces Kinks into alpha helix because it is unable to adopt normal helical shape and mostly reside in tight turns in protein structures. Although predicted conserved, Pro348 did not develop any type of interaction in CD-209 model, but three hydrophobic interactions come up with Trp343 and Trp327 by substituted Leu348 Fig 6.

#### W260C, W315R, W343G

**Fig 7:**
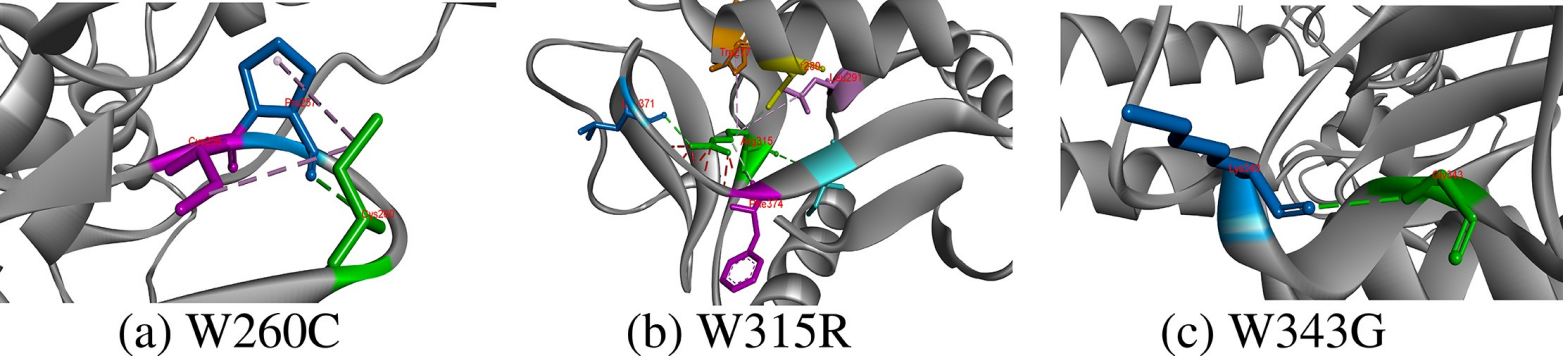
Hydrogen bond and other hydrophobic and electrostatic interactions created by substituted residues at 260, 315 and 343 positions in CD-209 model.

Tryptone is an aromatic and hydrophobic residue that prefers to be buried in protein hydrophobic core. It generally involves in stacking interactions with other aromatic side chain in protein structure. Total three hydrogen bonds with Pro257 and Trp258 and two hydrophobic interactions with Pro257 and Cys377 are produced by side chain of Trp260. Out of all interactions, only interactions with Pro257 were survived by replaced cysteine that also constituted an additional hydrophobic interaction with Cys256. Trp315 is an important residue at this position because it participated to create seven hydrogens bonds, four hydrophobic and one electrostatic interaction. Out of these hydrogen bonds of Trp315 with Phe374, Arg275, Ile376, Ser280 and Lys373, only four hydrogen bonds with Phe374, Ile376 and Ser280 and one extra bond with Leu371 were originated by side chain of substituted arginine residue. In addition, two hydrophobic interactions of substituted Arg315 with Leu291 and Trp277 also existed, which did not match with interactions formed by Trp315 with Lys373, Cys356 and Glu358. Lastly, Trp343 could only make one hydrogen bond with Lys340 along with five hydrophobic interactions with Trp327, Lys340 and Pro348. Unfortunately, when substituted Trp343, Gly343 only developed one hydrogen bond with Lys340 and broken all other hydrophobic interactions Fig 7.

## Conclusion

The role of missense SNPs leading to development of several diseases has always been under discussion demanding their rapid identification to understand the origin of pathologies. In literature, numerous missense SNPs in DC-SIGN receptor involved to capture the external intruders by interacting with their glycan moieties have reported that lead into causing HIV, dengue haemorrhage fever, etc. This research highlights the new missense SNPs snubbed in literature by their identification by using bioinformatics approach. Furthermore, it also exposes the structural position of substituted residues and damage by their replacement in term of energy stabilization and interaction to other residues. The paper can be a great interest for immune diseases specially caused by impairment of DC-SIGN receptor.

## Supporting information

S1 Table List of all 227 missense SNPs retrieved from Ensembl database and their impact by SNPs evaluating servers. (XLSX)
