# Correction: In silico screening and identification of deleterious missense SNPs along with their effects on CD-209 gene: An insight to CD-209 related-diseases

**DOI:** 10.1371/journal.pone.0271144

**Published:** 2022-07-05

**Authors:** Mohib Ullah Kakar, Muhammad Matloob, Rongji Dai, Yulin Deng, Kifayat Ullah, Ihsan Ullah Kakar, Ghulam Khaliq, Muhammad Umer, Zhoaib Ahmed Bhutto, Sarfarz Ali Fazlani, Muhammad Zubair Mehboob

There are errors in the author affiliations. The correct affiliations are as follows:

Mohib Ullah Kakar^1^, Muhammad Matloob^2^, Rongji Dai^1^ *, Yulin Deng^1^, Kifayat Ullah^3^, Ihsan Ullah Kakar^4^, Ghulam Khaliq^5^, Muhammad Umer^4^, Zhoaib Ahmed Bhutto^4^, Sarfarz Ali Fazlani^4^, Muhammad Zubair Mehboob

**1** Beijing Key Laboratory for Separation and Analysis in Biomedicine and Pharmaceuticals, School of Life Sciences, Beijing Institute of Technology (BIT), Beijing, PR China, **2** Department of Biomedical Engineering, National University of Science and Technology (NUST), Islamabad, Pakistan, **3** Department of Biosciences, COMSATS University Islamabad, Islamabad, Pakistan, **4** Faculty of Veterinary and Animal Sciences, Lasbela University of Agriculture, Water and Marine Sciences (LUAWMS), Uthal, Balochistan, **5** Department of Horticulture, Lasbela University of Agriculture, Water and Marine Sciences (LUAWMS), Uthal, Balochistan, **6** Department of Biochemistry and Biotechnology, University of Gujrat, Gujrat, Pakistan.
